# Thyroid dysfunction and Alzheimer's disease, a vicious circle

**DOI:** 10.3389/fendo.2024.1354372

**Published:** 2024-02-14

**Authors:** Zhaoqing Li, Jia Liu

**Affiliations:** Department of Thyroid Surgery, General Surgery Center, The First Hospital of Jilin University, Changchun, China

**Keywords:** thyroid dysfunction, hyperthyroidism, hypothyroidism, Alzheimer’s disease, cognition

## Abstract

Recently, research into the link between thyroid dysfunction and Alzheimer’s disease (AD) remains a current topic of interest. Previous research has primarily concentrated on examining the impact of thyroid dysfunction on the risk of developing AD, or solely explored the mechanisms of interaction between hypothyroidism and AD, a comprehensive analysis of the mechanisms linking thyroid dysfunction, including hyperthyroidism and hypothyroidism, to Alzheimer’s disease (AD) still require further elucidation. Therefore, the aim of this review is to offer a thorough and comprehensive explanation of the potential mechanisms underlying the causal relationship between thyroid dysfunction and AD, highlighting the existence of a vicious circle. The effect of thyroid dysfunction on AD includes neuron death, impaired synaptic plasticity and memory, misfolded protein deposition, oxidative stress, and diffuse and global neurochemical disturbances. Conversely, AD can also contribute to thyroid dysfunction by affecting the stress repair response and disrupting pathways involved in thyroid hormone (TH) production, transport, and activation. Furthermore, this review briefly discusses the role and significance of utilizing the thyroid as a therapeutic target for cognitive recovery in AD. By exploring potential mechanisms and therapeutic avenues, this research contributes to our understanding and management of this devastating neurodegenerative disease.

## 1 Introduction

The dysfunction of the endocrine system is increasingly associated with the pathological processes of Alzheimer’s disease and other forms of dementia (1, 2). The thyroid, which is a crucial endocrine gland, significantly determines the development of the central nervous system (CNS). Regulated by the hypothalamic-pituitary-thyroid axis, the primary function of the thyroid gland is to produce, store, and secrete thyroxine (T4) and a lesser amount of triiodothyronine (T3). Thyroid hormone (TH), functioning as a crucial neuromodulator, is critical to the regulation of neuronal differentiation, synaptic development, and myelination (3). Normal thyroid function is essential for growth, behavior, as well as mental and neurological development. Hyperthyroidism is characterized by an excessive production and release of TH. Hyperthyroidism affects multiple organ systems and typically manifests with symptoms like increased appetite with weight loss, heat-related symptoms, tremors, anxiety, fatigue, disrupted sleep, and palpitations (4). Hypothyroidism is a disease characterized by hypometabolism due to insufficient synthesis and secretion or physiological effects of thyroxine. In children, hypothyroidism is characterized by delayed growth and development, reduced physiological function, and cognitive impairment (5). Signs of hypothyroidism in adults include weight gain, intolerance to cold, fatigue, hoarseness of voice, dry skin, hormonal imbalances, and memory impairment (6). However, the clinical presentation of thyroid dysfunction can vary significantly based on factors such as age, gender, and the duration between onset and diagnosis.

Alzheimer’s disease (AD), often colloquially referred to as late-onset dementia, is a gradually advancing degenerative disorder that specifically targets the CNS. Tobore (2019) offers a quintuple framework encompassing all known risk factors, including thyroid dysfunction, vitamin D deficiency, sex hormones, mitochondrial dysfunction, and oxidative stress, each of them has a direct effect on the development and physiological processes of AD, both independently and in combination with each other, as well as through genetic interactions (7). Numerous studies have consistently demonstrated that the β-Amyloid peptide (βA) deposition of extracellular senile plaque and the phosphorylation tau of intracellular neurofibrillary tangles are significant factors contributing to the initiation and advancement of the disease, and in addition, activated glial cells and enlarged endosomes can be observed under the microscope (8). But more research still needs to be done. The biological processes underlying neurodegeneration in Alzheimer’s disease are still hugely controversial, including other protein components that co-aggregate with βA, loss of synaptic homeostasis, clearance of damaged proteins, and network connectivity in the cortex (8, 9). Moreover, early diagnosis of the disease is a major challenge. These include the ambiguity of early diagnostic criteria, the limitations of diagnostic tools, and the fact that other neurodegenerative or cerebrovascular diseases can also cause early mild cognitive impairment (8). And the slow pathological changes in AD also make our early diagnosis difficult (10). Furthermore, the neurotoxic properties of βA and p-tau, along with their prion-like transmission nature, enable them to spread between neurons, sowing cytotoxic seeds that propagate cytotoxicity and exacerbate the pathological changes that characterize AD (11).

An increasing body of evidence suggests a strong link between thyroid dysfunction and Alzheimer’s disease (2, 3). Therefore, by analyzing the role of thyroid dysfunction as a potential cause and consequence of AD, as well as using the thyroid as a therapeutic target for treating AD, our objective is to gain a thorough understanding of the causal connection between thyroid dysfunction and AD. Additionally, we propose a concept of a “vicious circle” to describe this relationship, highlighting the reciprocal influence between the two conditions. We anticipate that these findings will lay the foundation for the creation of individualized and innovative treatment approaches for individuals who are impacted by AD.

## 2 The relevance between thyroid dysfunction and AD

The association between hyperthyroidism and heightened susceptibility to AD has been a subject of debate in the academic community. A cohort study conducted in 2006 by de Jong, den et al. challenged the idea that thyroid function significantly influences the onset of AD (12). Similarly, a 3-year follow-up study involving individuals over 85 years old concluded that thyroid function status has no impact on cognition (13). Another cross-sectional study conducted on 295 Chinese elderly patients with cognitive impairment, as reported by Hu, Wang et al. in 2016, observed no significant link between hypothyroidism or subclinical hyperthyroidism and AD or mild cognitive impairment (MCI) (14). Annerbo highlighted that low TSH levels are strongly linked to AD patients, but suspected that homocysteine ​​rather than TSH might be associated with the development of AD and homocysteine was negatively correlated with TSH (15). Furthermore, a bidirectional two-sample Mendelian randomization study (16) and a meta-analysis (17) both concluded that there is no causal association between hyperthyroidism and AD. However, a study in 2010 found that although there was no association between TT3 and cognition in the control group, patients with MCI exhibited a negative relationship between serum TT3 levels and cognitive function. This implies that higher TT3 levels, combined with increased AD pathological changes, might contribute to a deterioration in cognitive function (18).

Some findings have also indicated that elevated levels of T4 are linked to an elevated likelihood of developing dementia and AD (19, 20). According to a longitudinal study conducted by Yeap, higher circulating levels of fT4 are an independent predictor of dementia in elderly men (21). At the same time, other researches have shown a closer linkage between lower TSH concentrations and a higher risk of AD. In comparison with individuals having normal TSH levels, a decrease in TSH by 6 months is linked to a 16% higher risk of developing dementia (22). A meta-analysis of cohort studies involving 344 248 individuals revealed that only subclinical hyperthyroidism exhibited a connection with an increased risk of dementia, with TSH concentrations ranging from 1.55-1.60mU/L being associated with the highest risk (23). In contrast, subclinical hypothyroidism, clinical hyperthyroidism and clinical hypothyroidism had no effect on dementia (23). In 2000, Kalmijn et al. conducted a prospective study focused on subclinical hyperthyroidism in the elderly, specifically examining its association with dementia and AD. Interestingly, after accounting for potential confounding factors such as age, gender, and atrial fibrillation, the study discovered that this correlation persevered and held substantial weight (24). Additionally, it was observed that individuals with positive TPO-Abs (Antibodies to Thyroid Peroxidase) coexists with low TSH levels were particularly vulnerable to developing dementia, which reminds us of the importance of autoimmune thyroid disease in AD (24).

Research has consistently demonstrated a strong link between hypothyroidism and an elevated likelihood of developing AD. Significant hypothyroidism can impair various cognitive functions, including thinking, speech, attention, concentration, general intelligence, perceptual function, psychomotor function, and executive function (25). Specifically, memory experiences the greatest impact (25). A reanalysis of eight case-control studies exposed a significantly heightened risk of AD in patients with hypothyroidism (26). Similarly, Quinlan’s prospective study in 2014 discovered an inverse correlation between lower levels of serum FT3 and the risk of AD development, and comparatively individuals in the lowest quartile of FT3 levels exhibited more than twice the risk of subsequently developing AD in comparison to those in the highest quartile (27).

We present our perspective on the ongoing debate surrounding the relationship between thyroid dysfunction and the risk of developing Alzheimer’s disease. One of the key issues in the research is the use of inadequate sample sizes and inappropriate proportions (13, 17, 20). Insufficient total sample size and a small proportion of the population with thyroid dysfunction can result in less accurate study effect estimates. Another important factor to consider is the limitations of the study methodology. In cross-sectional studies, it is challenging to determine the chronological order of thyroid dysfunction and Alzheimer’s disease (14, 18). Moreover, most prospective studies only assess thyroid status once during the follow-up period, without tracking any changes in the participants’ thyroid function (12, 13, 24). This lack of information regarding spontaneous normalization of thyroid function or other interventions can impact the study’s effectiveness. Additionally, the absence of sufficient follow-up time and imprecise assessment of cognitive status during follow-up are areas that require improvement and further development (12, 24). Misclassification of thyroid function is also a potential concern, as thyroid hormone requirements vary based on age, gender, and race (15, 17, 19, 20). More critically, few measurements of CNS levels of TH are currently available. Another challenge lies in the unclear diagnosis of Alzheimer’s patients (20). Most studies solely rely on cognitive status assessment and clinical presentation without a clear pathological diagnosis, making it difficult to determine whether individuals with thyroid dysfunction have developed Alzheimer’s disease pathology without cognitive impairment after several years of follow-up. It is also unclear whether pathological changes associated with Alzheimer’s disease were already present before the onset of thyroid dysfunction, and cognitive function had not yet been affected. In conclusion, future studies should focus on appropriate sample sizes and proportions, accurate diagnosis of thyroid functional status, and precise prospective studies for the diagnosis of Alzheimer’s disease.

## 3 Thyroid dysfunction as the result of AD

Viewed from the thyroid status in the early stages of AD, it appears that there is an increased risk of hyperthyroidism. Ren et al. created an early Alzheimer’s disease (AD) rat model by injecting okadaic acid (OA) into the rats’ hippocampus, which activated GSK-3, increased higher oxidative stress, generated tau hyperphosphorylation, and increased βA neurotoxicity. The study’s findings demonstrated a notable increase in FT3, FT4, TSH, and TRH levels within the brain tissues of rats in the early stages of AD, and there was a notably higher expression of TH receptors in the hippocampal tissues compared to the control group, along with FT4, TSH, and TRH were up in blood, with no change in FT3 (28). As a result, it came up that the hyperthyroid condition observed in the brain and circulation during early AD could be an early local and systemic stress repair response. Furthermore, Quinlan et al. discovered that elevated serum T4 levels in relatively early AD were not accompanied by elevated T3 levels, and that these patients did not have thyroid dysfunction or use drugs that could alter TH levels, which is consistent with Ren’s findings (29). These findings imply that peripheral T4 to T3 conversion is decreased in early AD. It remains unclear whether this result is due to a self-protective mechanism of high serum T4 in early Alzheimer’s disease or a result of lower deiodinase activity during the primary pathological process in AD patients. Unfortunately, both Ren and Quinlan et al. failed to detect and analyze deiodinase, despite both acknowledged its importance in this process. Therefore, the levels of deiodinase in the circulation and brain tissue of patients with early Alzheimer’s disease need to be further investigated.

As the pathogenic process of AD worsens, there is a continuous accumulation of toxic βA. The development of a hypothyroid state is linked with the disease’s progression to the middle and late stages. According to our viewpoint, the loss of compensation for the stress repair response and the expanding lesion are two essential factors that contribute to this. In the first study exploring the hypothalamic-pituitary-thyroid axis in AD, the levels of TRH, TSH and TH were significantly lower in patients with AD (30). In the AD cases, there were no significant correlations observed between TRH and TSH, TT3, TT4, FT3, and FT4, as well as between TSH and TT3, TT4, FT3, FT4 (30). However, in the healthy controls, TRH showed significant correlations with TSH and FT4, while TSH exhibited significant correlations with TT4 and FT4 (30). This shows a severe deterioration of the biofeedback regulation of hypothalamic-pituitary-thyroid axis. Similarly, Chen et al. discovered that serum TSH levels in AD patients lacked a circadian rhythm and were significantly lower than health controls (31). Hence, we speculate that diminished TRH and TSH secretion as well as the absence of normal feedback control caused by degeneration and impaired function of the hypothalamus and pituitary gland, result in the development of hypothyroidism in patients with AD during the mid to late stages. Insufficiently, to our knowledge, as the studies mentioned above have mostly analyzed the function of the hypothalamic-pituitary-thyroid axis in patients with Alzheimer’s disease through changes in the levels of thyroid hormones and related hormones, and there is a lack of definitive pathological diagnosis of definite hypothalamic and pituitary degeneration. In the future, a clear pathological diagnosis of hypothalamic and pituitary degeneration during disease progression in AD is needed.

As Alzheimer’s disease progresses, TH transport mechanisms may be also disrupted. A huge calcium inflow is caused by increased ryanodine receptor expression in AD patients, activating the nNOS expression in neurons and leading to overproduction of NO, which directly accelerating the evolution of the AD pathology and suppresses monocarboxylate transporter 1 (MCT1) expression in both neurons and glial cells (32). We speculate that the same mechanism may also apply to the thyroid hormone transporters MCT8 and MCT10, but unfortunately no evidence has yet been found to confirm our speculation. TTR (transthyretin) is a homotetrameric protein, with four identical subunits, the liver secretes it into the bloodstream while the choroid plexus secretes it into the cerebrospinal fluid (33). When toxic pathological products of AD disrupt the structure and function of the choroid plexus, the concentration of TTR in the brain decreases. TTR is responsible for the transfer of T4 from the bloodstream to the cerebrospinal fluid, thus ensuring that T4 is not lost across the blood-brain barrier and maintaining an optimal concentration of T4 in the brain (33). However, it has also been suggested that TTR was not necessary for the upkeep of thyroid hormone homeostasis (34). TTR has been reported to function as a pro-angiogenic agent during early stage AD development by upregulating the expression of VEGF, Ang-2, IL-6 and IL-8 (33). Meanwhile, TTR can also regulate βA metabolism by inhibiting βA production, accelerating βA clearance and attenuating βA toxicity (35). Therefore, the stability of TTR function is essential for the whole pathological process of AD and plays a key neuroprotective role. However, it remains unsatisfactorily investigated whether TH transport is impaired in AD. First, the mechanism of MCT1 downregulation during AD pathology is uncertain as to whether it applies to MCT8 or MCT10. Second, the role of TTR in maintaining TH concentrations in the brain is controversial. Hence, we are looking forward to further studies on TH transport during AD pathology.

Deiodination is the initial stage in the activation or inactivation of thyroid hormone, and deiodinases are important in this process (36). The exocyclic deiodination of T4 and its conversion to the active T3 are the primary reactions catalyzed by iodothyronine deiodinase 1 (DIO1) and iodothyronine deiodinase 2 (DIO2), which promote the activation pathway. In the inactivation pathway, iodothyronine deiodinase 3 (DIO3) acts as an essential endocyclic deiodinase by catalyzing the conversion of active T4 and T3 to inactive reverse-T3 and T2 (36). DIO2 and DIO3 are deiodinases that function principally in the central nervous system and are subcellularly localized in endoplasmic reticulum membranes and cell membranes, respectively (36). Based on a whole transcriptome study, a significant transcriptional difference specific to late-onset AD was found, in which the expression of DIO2 was reduced (37). The high oxidative stress in Alzheimer’s patients also decreases DIO2 activity and increases DIO3 activity (36). Wajner et al, by modelling deiodinase dysfunction under IL-6-induced oxidative stress, found that after partial correction of the oxidative stress state by n-acetylcysteine supplementation, DIO3 activity was fully restored, whereas DIO2 or DIO1 function was not (38). Therefore, how oxidative stress destroys deiodinase function, and whether there are etiological factors other than oxidative stress, for deiodinase dysfunction that occurs during the pathological process of Alzheimer’s disease are directions that need to be explored and investigated more in the future.

**Figure 1:**
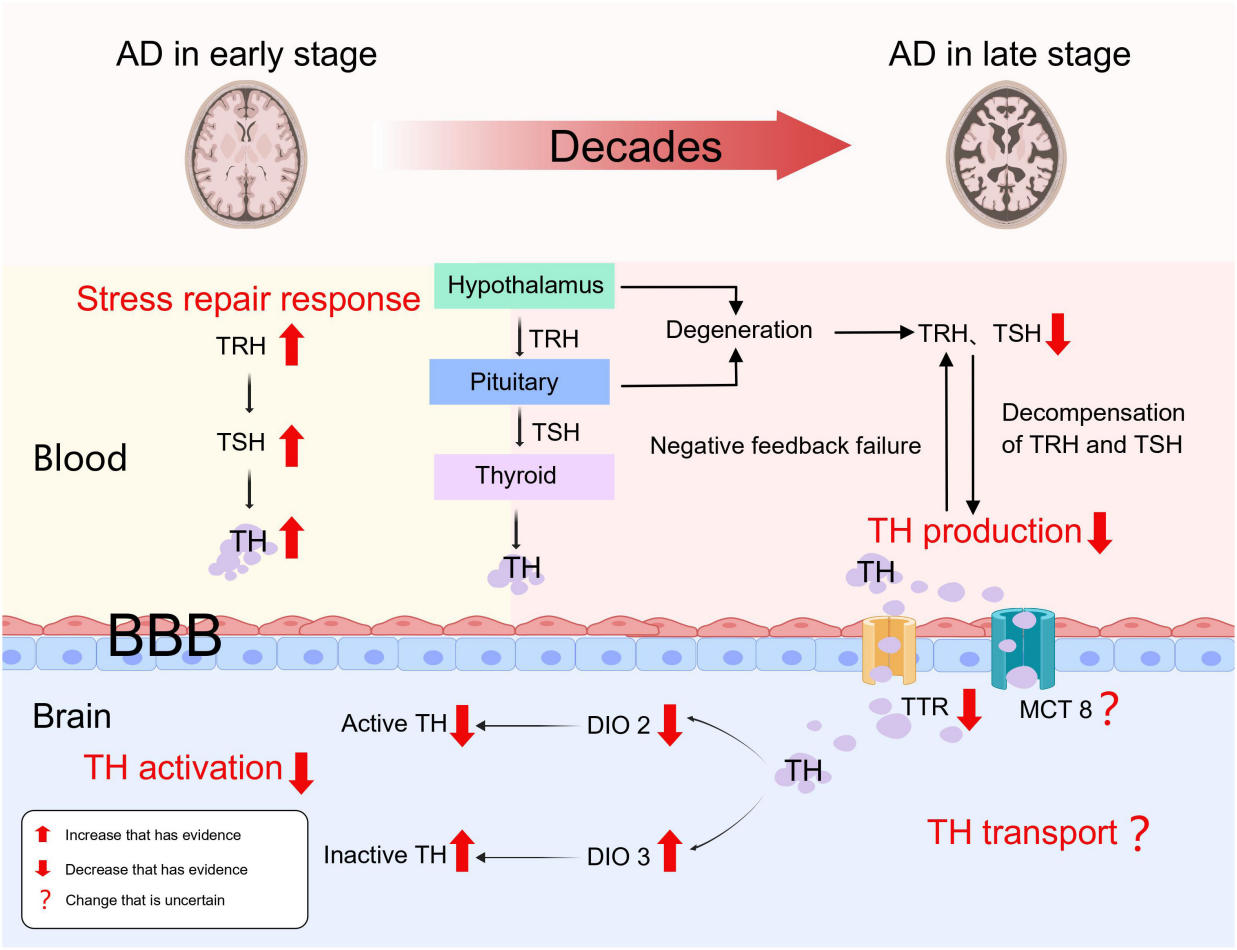
Thyroid dysfunction as a result of AD.

In our conclusion but not a proven fact, as the pathophysiological process of AD progresses, the functional state of the thyroid gland undergoes changes. It can be divided into two stages: early hyperthyroidism due to stress repair response, and a mid to late phase hypothyroid state caused by abnormalities in TH production, transport, and activation (**Figure 1**).

## 4 Thyroid dysfunction as the cause of AD

### 4.1 The effect of hyperthyroidism on AD

The underlying processes of the relationship between hyperthyroidism and Alzheimer’s disease must be thoroughly studied. According to an autopsy in 2009, more neurofibrillary tangles and neocortical plaques were found in patients having higher total thyroxine levels (39). For the first time, Li, Yang et al. discovered in 2020 that patients with hyperthyroidism exhibited notably elevated levels of total serum tau in comparison to those with normal thyroid function (40). These observations serve as a reminder that tau protein aggregation and phosphorylation are hallmarks of an AD pathological process that may be present in hyperthyroid patients. But unfortunately, in these studies, we were not able to find a causal relationship between high thyroid hormone levels and the development of AD. More importantly, plasma Tau levels only partially reflect the pathological process of AD and their diagnostic accuracy is poor compared to cerebrospinal fluid Tau levels (41). We look forward to finding more sensitive, specific and easily sampled biomarkers for AD in the future.

At the cellular level, Hyperthyroidism coexists with a lipid peroxidation state which can be inhibited by treatment of hyperthyroidism (42). In addition, liver mitochondria in the hyperthyroid state have a strong ability to scavenge H_2_O_2_, resulting in a substantial production of reactive oxygen species (ROS) and damaging redox equilibrium (43). However, these findings only demonstrate the high oxidative stress state of hyperthyroidism from peripheral tissues and do not prove whether the same mechanism applies to the central nervous system. Nonetheless, it is clear that correcting the hyperthyroid state improves the state of oxidative stress throughout the body (44). And the use of antioxidants can reduce the oxidative stress and neuronal cell death in the hippocampus of the brain caused by hyperthyroidism (45). Oxidative stress aggravates the pathogenic process of AD by promoting the accumulation of βA, hyperphosphorylation of tau, and the loss of synapses and neurons (46). A vicious loop is created when abnormal βA or phosphorylated tau protein builds up, further promoting redox imbalance (47). More crucially, when the amount of ROS produced in some neurodegenerative diseases—like Alzheimer’s disease—is too high, the delicate balance that fragile neurons maintain is more likely to be upset, which increases the likelihood of synapse loss and dendritic damage, ultimately resulting in neuronal death and axonal pathology (48). In addition, the excessive presence of thyroid hormone hampers the neuronal proliferation, regeneration and repair processes by impeding the expression of DNA synthesis and cell cycle-related antigens in the region of proliferating cells within the CNS (49). In a newly published study, hyperthyroidism exacerbates cognitive impairments and increases the accumulation of βA plaques in mice by the activation of neuroinflammation and the induction of brain tissue necroptosis via the RIPK3/MLKL pathway (50).

In addition, elevated thyroid hormone can cause the atrophy of hippocampal and temporal lobe structures (12). According to research findings, the administration of excessive thyroid hormone in young rats has been observed to lead to a notable reduction in long-term potentiation (LTP), deficits in cognitive functions, as well as significant neurochemical and morphological changes in the hippocampus (51). As reported, T4 has the ability to trigger long-term depression (LTD) and reduce LTP through a process known as non-genomic, membrane priming effect (52). But the molecular mechanisms of how TH mediates LTD and LTP await further anatomical and pharmacological analysis. It was also found that people diagnosed with hyperthyroidism have a reduced functional connectivity between the hippocampus and the cerebral cortex (53). In hyperthyroid mice, a reduction in the density of mature dendritic spines in the hippocampus has been observed, accompanied by impaired function of postsynaptic α-Amino-3-hydroxy-5-methyl-4-isoxazole propionic acid receptors (AMPAR) and N-methyl-D-aspartate receptors (NMDAR), leading to decreased excitatory synaptic transmission (54). Also, the expression of NMDAR subunit GRIN2B gene is downregulated in the hippocampus tissue, which affects spatial memory that depends on the hippocampus (55). Moreover, hyperthyroidism has been found to potentially result in a decline in brain-derived neurotrophic factor (BDNF) levels (56). As a result, patients with hyperthyroidism may face difficulties in learning tasks that heavily rely on the hippocampus and are prone to forgetting them at a faster rate. Additionally, as TRH levels drop, the MAPK pathway’s signal weakens, releasing its inhibition on GSK3-activity and causing tau to become hyperphosphorylated (57).

The brain metabolism of patients with hyperthyroidism is also significantly altered, with diffuse and global neurochemical disturbances. A seminal study conducted the first functional brain imaging investigation in individuals with thyrotoxicosis, revealing a noteworthy decrease in glucose metabolism within the limbic system, a brain region crucially involved in the consolidation of long-term memory (58). According to Danielsen et al., it was observed that there was a sustained disturbance in the glutamate-glutamine cycle within the cerebral white matter in cases of hyperthyroidism, and additionally, the levels of total choline and myo-inositol were significantly lower during the acute phase of hyperthyroidism compared to the control group (59). Also, the importance of TRH for the significant and long-lasting release of endogenous acetylcholine in the hippocampus and the increased acetylcholinesterase activity in hyperthyroidism remind us of the importance of hyperthyroidism on the metabolism of acetylcholine (60, 61). These studies suggest that there may be a loss of acetylcholine due to TRH depletion, the elevation of acetylcholinesterase activity or other reasons yet to be explored in the hyperthyroid state, leading to cognitive impairment. However, to our knowledge, no study has confirmed the reduction of acetylcholine in the hyperthyroid state. What’s more, the activation of the thyroid hormone response system has been observed to result in the upregulation of neuroserpin in the brain, which is the primary inhibitor of tissue plasminogen activator within the brain, and its upregulation leads to the deposition of βA in the brain (62). Besides, a nationwide study in Spain showed that hyperthyroidism increases the risk of cardiovascular disease (63). Meanwhile, the impact of cardiovascular disease on AD can be explained by the “two-hit hypothesis”, namely reduced cellular blood flow and blood-brain barrier dysfunction (64). These two events have a synergistic role in the accumulation of βA in the brain, which leads to neuronal loss and cognitive decline.

**Figure 2:**
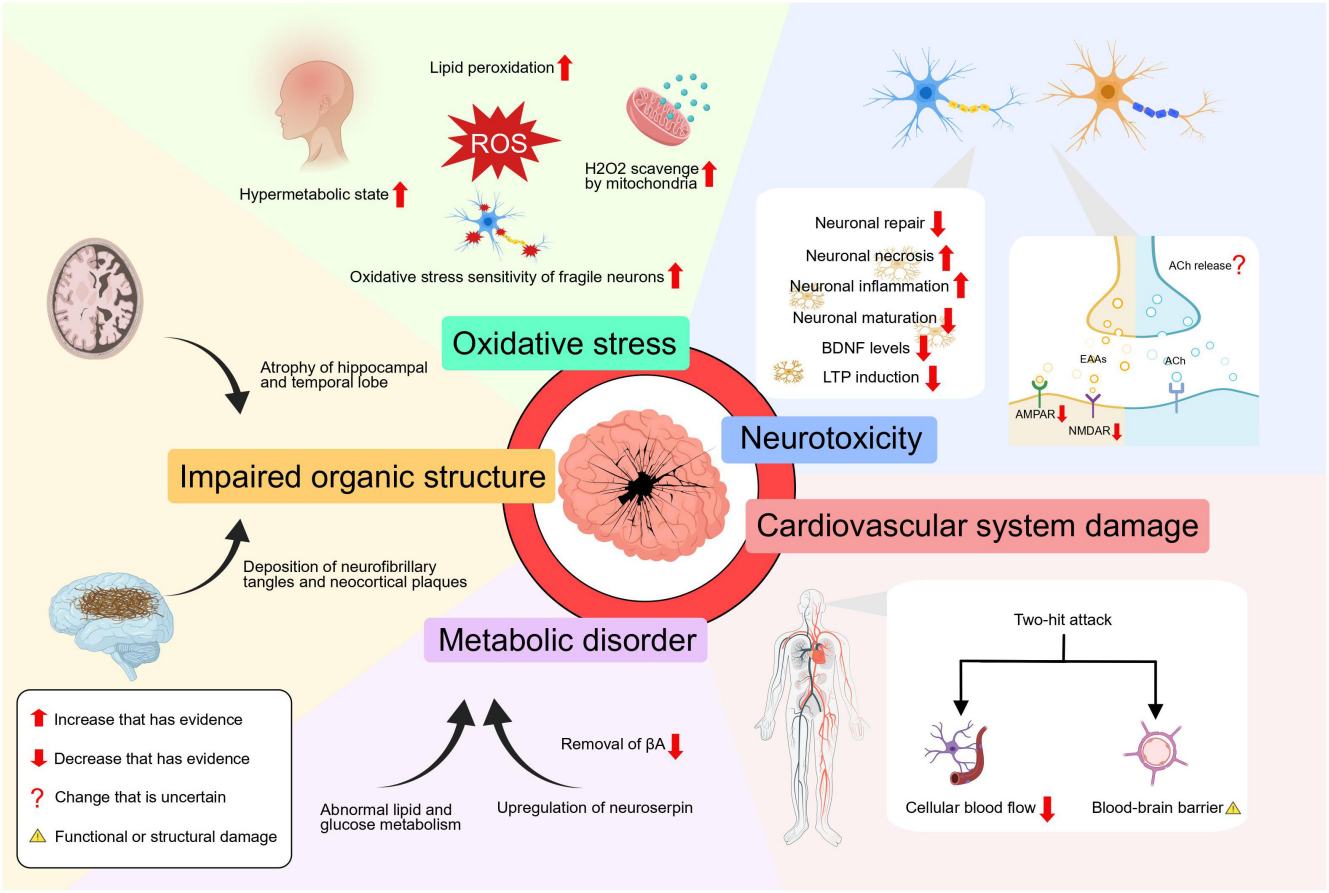
The effect of hyperthyroidism on AD.

It is worth noting that these aforementioned influences do not exist in isolation, but rather interact and mutually affect each other. And we must emphasize the lack of convincing conclusions between hyperthyroidism and AD. By our speculation, hyperthyroidism could disturb the structure and function of neurons and the hippocampus, disturb the redox balance, alter the metabolic state of the brain, upregulate the expression of serine proteases, and even exposes CNS to a “two-hit” attack through the cardiovascular system. These concurrent mechanisms may all be involved in the pathogenesis of Alzheimer’s disease (**Figure 2**).

### 4.2 The effect of hypothyroidism on AD

Thyroid hormone acts as a neuromodulator and crucially impacts mood regulation, neurotransmitter function and regulation, brain development and function, neuroprotection and brain metabolism. The hippocampus displays a high concentration of receptors for TH and dysregulation of TH results in significant impairment of both cognitive and memory functions (65). Thyroid hormone enhances the action of nerve growth factor and promotes the growth of neurites (66). Thyroid hormones can also affect adult hippocampal neurogenesis by stimulating the proliferation, survival and differentiation of dentate granule cell progenitors (67). Expression of significant components in the MAPK/ERK-CREB and Ca2+/Calmodulin pathways, which are crucial for synaptic plasticity, decreases in the hypothyroid state and restored after thyroid hormone supplementation (68). Meanwhile, antithyroid drug administration led to a reduction in serum BDNF levels and a developmental delay in primary hippocampal neurons in rats, and the addition of BDNF can rescue this variation (69). Input-output connections and LTP of the perforant pathway were greatly reduced in animals with hypothyroidism, and the ability of synapses between the perforant pathway and the dentate gyrus to undergo LTP was significantly impaired (70).

Hypothyroidism can lead to impaired glutamatergic synaptic function. On one hand, the release of the excitatory neurotransmitter glutamate is decreased (71) and there is also less expression of the postsynaptic neuronal NMDAR subunit NR1 mRNA in the hippocampus (72). This decrease causes a block and delay in synaptic transmission, it also inhibits the proliferation and differentiation of neural stem cells (73). On another, the reduction in thyroid hormone makes neurons more sensitive to the neurotoxic effects of glutamate, which may be attributed to the ability of thyroid hormone in regulating extracellular levels of glutamate by modulating transporter proteins on astrocytes (74). Hypothyroidism causes abnormalities in cholinergic neurons, with significant reductions in hippocampal acetylcholine content and choline acetyltransferase activity, and the cholinergic neurons exhibit abnormal electrophysiological properties including depolarization of resting membrane potentials and elevated thresholds (75–77). Also, evidence from many studies has shown the importance of thyroid hormone supplementation combined with the use of cholinesterase inhibitors in reversing synaptic damage and restoring cognitive function (75, 78).

Hypothyroidism facilitates AD via APP (Amyloid Precursor Protein) and TREM2 (Triggering Receptor Expressed on Myeloid Cells 2) gene expression. Preclinical research indicates that thyroid hormones block APP gene expression by reducing histone H3 acetylation and histone H3 lysine 4 methylation, and thus reduce βA formation (79). Regulation of TREM2 gene expression on microglia by thyroid hormones has also been demonstrated (80). If TREM2 dysfunction occurs, it may alter the response of microglia to βA and may be involved in the etiology of Alzheimer’s disease through immune and inflammatory pathways (81). Hypothyroidism also leads to an increase in pro-inflammatory factors such as IL1β, IL6 and TNF expression within the hippocampus (68). Activation of interleukin 1 (IL-1) prompts autophagy, which in turn, triggers the death of brain cells in the hippocampus resulting in impaired cognitive function in young rats (82). At the same time, autophagic vesicles possess the necessary components for βA synthesis, and the exaggerated autophagic pathway contributes to βA accumulation (83). Furthermore, research has also shown that hypothyroidism can upregulate autophagy and apoptosis in neurons of the cerebellum, and the autophagic process can be attenuated by thyroid hormone replacement (84). One study summarized the relevance of autophagy failure to ageing and AD, while suggesting the possibility of stimulating autophagy in AD therapy (85, 86). This suggests to us that the autophagy pathway may be overwhelmed by the accumulation of oxidative stress and cellular damage, resulting in relative autophagic insufficiency. How to effectively regulate autophagic activity may become a potential treatment for AD and other neurodegenerative diseases.

Hypothyroidism can induce damage to the hippocampus and trigger oxidative stress states by activating endoplasmic reticulum stress (87). Meanwhile, endoplasmic reticulum stress is a critical step in multiple neurodegenerative pathologies that are marked by the buildup of misfolded proteins and aggregates (88). Due to the important function of which thyroid hormones play in mitochondrial function and energy metabolism, mitochondrial dysfunction may also occur in thyroid hormone deficiency (89). Damage to mitochondria may increases the vulnerability of neurons to oxidative stress (90). And oxidative stress and mitochondrial dysfunction have key roles in the pathogenesis and progression of AD (91). In brain metabolism, thyroid hormone enhances insulin signaling in the hippocampus, decreases activation of glycogen synthase kinase 3 (GSK3) and tau protein levels of the hippocampus, and slows neurodegeneration (92). Age-related hypothyroidism increases transportation of APOE4-loaded exosomes from the liver to the brain, ultimately leading to disrupted lipid metabolism in the brain, accelerated accumulation of ROS and promotion of neuronal cell death (93). Meanwhile, new study in mouse model with subclinical hypothyroidism has demonstrated the possible participation of endoplasmic reticulum stress in lipid metabolism disorders (94). Cerebral blood flow to brain regions associated with memory is lower in patients with subclinical hypothyroidism and mild hypothyroidism (95, 96). Long-term inadequate blood flow to the brain specifically lowers neuronal activity in the limbic system, damages cognitive memory, and participates in the abnormal accumulation of βA and p-Tau that occur in AD (97).

**Figure 3:**
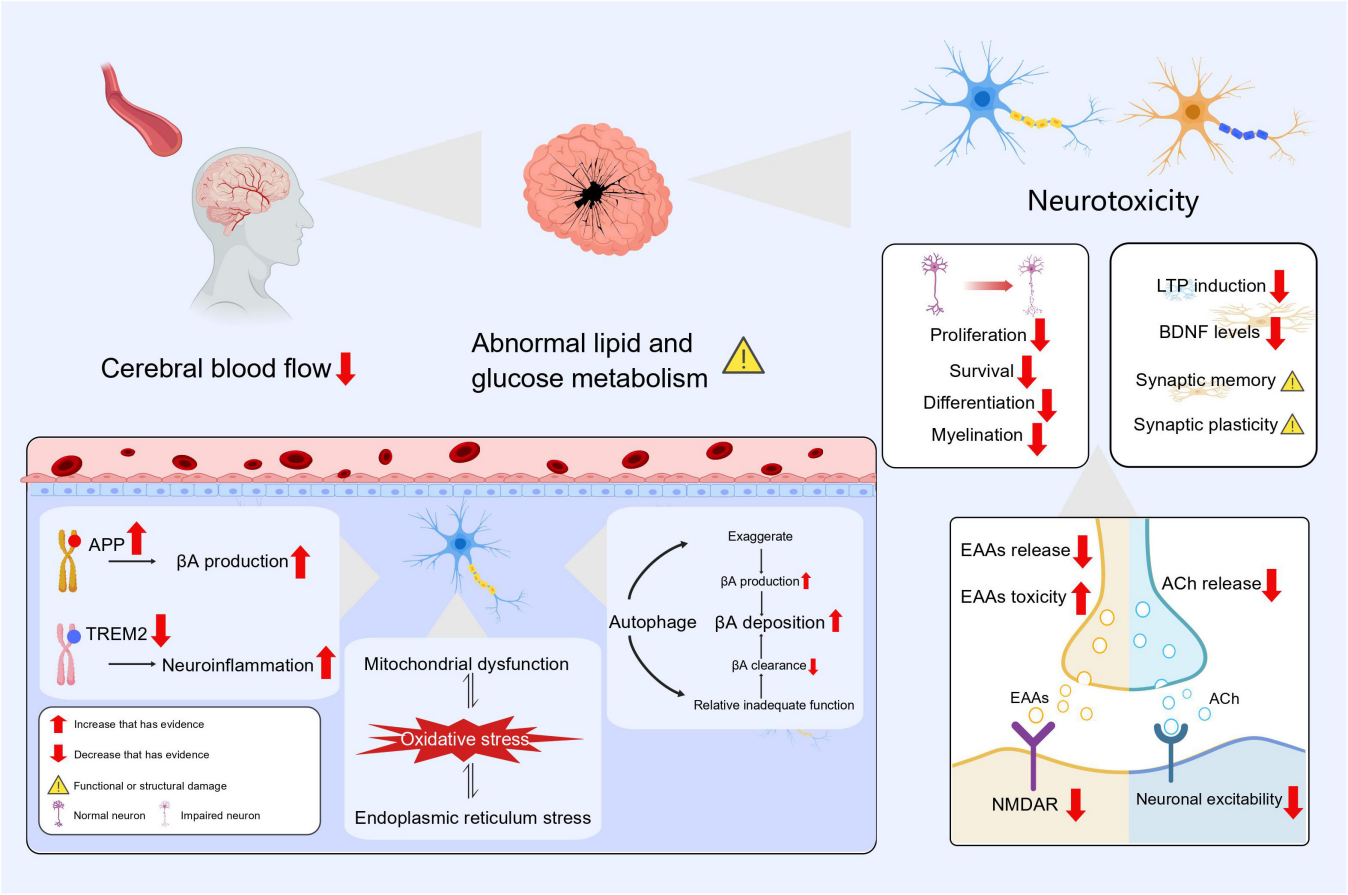
The effect of hypothyroidism on AD.

As shown above, the impact of hypothyroidism on AD can be examined through various mechanisms, including diminished neuromodulation, alterations in gene expression, impaired autophagy, elevated oxidative stress, and cerebral metabolic dysfunctions (**Figure 3**). It should be noted that these elements do not operate independently, but instead collaborate synergistically to foster the onset and advancement of AD. And we must point out that these are only possible mechanisms for the link between hypothyroidism and Alzheimer’s disease. The exact mechanisms for the connections between the two are inconclusive and need to be determined in future studies.

## 5 Thyroid as a target for AD therapy

In recent years, many studies have highlighted the significance of antithyroid therapy in enhancing cognitive function. A systematic review encompassing 31 high-quality studies conducted over the past two decades, focusing on the quality of life of patients with primary hyperthyroidism following thyroidectomy, strongly indicates that treatment of hyperthyroidism leads to a significant and enduring improvement in neurocognitive status (98). Antithyroid medication improves learning ability in individuals with hyperthyroid dementia, and parts of the frontoparietal lobe with uptake impairments gradually respond to the medicine (99). However, antithyroid therapy does not completely reverse the symptoms of dementia that occur in hyperthyroid patients. Kumar et al. discovered that, by examining changes in key metabolites in the posterior parietal cortex and the dorsolateral prefrontal cortex in hyperthyroid patients with an overactive thyroid before and after receiving medication to treat the condition, the proportion of neuro-metabolites in brain regions, while reversible, was not completely reversed even when normal thyroid function was achieved (100). Meanwhile, although functional brain connections and cognitive abilities were improved in hyperthyroid patients treated with antithyroid therapy, they were not totally recovered (101).

Similarly, proper care of hypothyroid patients is critical. Thyroid hormone supplementation therapy can reverse memory deficits and alleviate cognitive deficits by reducing βA production, decrease neuroinflammation, stimulating synaptic plasticity and memory enhancement, and restoring key indicators of cell signaling pathways in the hippocampus (68). Thyroid hormone replacement treatment is required for the restoration of hippocampus impairments caused by hypothyroidism (102). However, similar to the response to antithyroid therapy, the response to thyroid hormone replacement therapy is not complete or sufficient. Farag et al. found that thyroid hormone supplementation has the ability to repair and preserve synapses and dendrites within the hippocampus, but they also noted that this regeneration is insufficient and incomplete (103). Yet, that study only demonstrated histological hippocampus recovery and did not include additional functional and neurophysiological assessments (103). Meanwhile, a systematic review of thyroid hormone replacement therapy for cognitive dysfunction discovered that thyroid hormone benefits in the treatment of cognitive deficits resulting from hypothyroidism, but that this therapeutic effect is not fully and always effective, and may even result in side effects (104).

With the above, it can be seen that the management and treatment of patients with hyperthyroidism or hypothyroidism can partially ameliorate and alleviate symptoms, although it does not completely normalize cognitive function altogether. The incomplete recovery of cognitive function in patients after antithyroid therapy or thyroid hormone replacement may be attributed to several factors. Firstly, the multifaceted nature of the etiology of Alzheimer’s disease and the complexity of the pathology contribute to this issue (7, 8). Secondly, individual differences among patients, such as age, gender, genetics, lifestyle, the presence of underlying diseases, and even the duration of the abnormal thyroid status, also play a role. Thirdly, the limited action of thyroxine and the uncertainty of thyroxine levels in the brain., particularly the regionalized effects of thyroid hormones on brain metabolism and selective differential contributions to hippocampal circuitry and function, have been observed (79, 100). And in some studies, there was a slight improvement in serum thyroid hormone levels, but brain thyroid hormone levels were not re-established (103). Lastly, the limited sample size and insufficient follow-up period in previous studies suggests whether inadequate recovery is related to insufficient treatment duration (100, 101). Further research is needed with larger sample sizes and longer durations, including different time points for evaluation. In the future, normalization of thyroid status in combination with other interventions such as antioxidants, cholinesterase inhibitors, stabilization of neuronal calcium ions and modulation of autophagy have strong therapeutic potential for neurodegenerative diseases such as AD (45, 78, 83, 105).

Even in cognitively normal subjects with an abnormal brain βA burden, it usually takes more than 10 years before cognitive decline begins (10). More critically, maintaining homeostasis of thyroid hormone levels within the brain is critical to the proper functioning of the CNS, and even relatively minor deviations in brain hormone levels can cause severe behavioral and cognitive impairment (106). Therefore, it is vital to provide suitable therapy at the best time for each individual patient to prevent and treat cognitive deficits caused by thyroid dysfunction.

## 6 Conclusion

In this discussion, we have provided a detailed analysis of the interrelationship between thyroid dysfunction and Alzheimer’s disease (AD). However, we must admit that the exact mechanisms and the sequence of events between thyroid dysfunction and AD have not yet been convincingly concluded. We require longer-term, large-scale clinical trials and more in-depth basic research at various levels, including molecular, cellular, and animal models. Meanwhile, methods for measuring thyroid hormone levels in the central nervous system, more appropriate criteria for assessing thyroid function in specific populations, and more sensitive, specific and convenient diagnostic methods for Alzheimer’s disease need to be further explored. We look forward to more in-depth studies in the future to gradually improve the pathological mechanisms between thyroid dysfunction and Alzheimer’s disease, and to find a more comprehensive and convincing network of mechanisms. Such efforts will help develop more effective clinical treatment strategies. Moreover, it is crucial to prioritize early detection, diagnosis, and treatment by assessing thyroid function in individuals at a high risk of developing neurodegenerative diseases. This approach could significantly contribute to the prevention and deceleration of Alzheimer’s disease progression.

## Author contributions

ZL: Writing – original draft. JL: Writing – review & editing.
